# High preoperative albumin-bilirubin score predicts poor survival in patients with newly diagnosed high-grade gliomas

**DOI:** 10.1016/j.tranon.2021.101038

**Published:** 2021-02-14

**Authors:** Jie Zhang, Qiuyan Xu, Hua Zhang, Yihong Zhang, Yu Yang, Huidan Luo, Xiaoyan Lin, Xingqin He, Yonggao Mou, Zhihuan Zhou, Zhenqiang He

**Affiliations:** Department of Neurosurgery/Neuro-oncology, Sun Yat-sen University Cancer Center, State Key Laboratory of Oncology in South China, Collaborative Innovation Center for Cancer Medicine, Guangzhou 510060, China

**Keywords:** High-grade gliomas, Albumin-bilirubin score, Tumor progression, Prognosis, Survival

## Abstract

**Objective:**

To determine the prognostic value of the preoperative Albumin-bilirubin (ALBI) score in high-grade glioma (HGG) patients.

**Methods:**

A retrospective study of 194 HGG patients was conducted. ROC analysis was used to determine the optimal cut-off value of ALBI score. Univariate and multivariate analysis was performed to identify prognostic factors associated with progression free survival (PFS) and overall survival (OS). The resulting prognostic models were externally validated by a demographic-matched cohort of 130 HGG patients.

**Results:**

Optimal cutoff value of ALBI score was -2.941. In training set, ALBI was correlated with age (*P* = 0.001), tumor location (*P* = 0.012) and adjuvant therapy (*P* = 0.016). Both PFS (8.27 vs. 18.40 months, *P*<0.001) and OS (13.93 vs. 27.57 months, *P*<0.001) were significantly worse in the ALBI-high group. Strikingly, patients in ALBI-low group had 56% decrease in the risk of tumor progression and 57% decrease in the risk of death relative to high ALBI. Multivariate analysis further identified ALBI score as an independent predictor for both PFS (HR=0.47, 95% CI 0.34, 0.66) and OS (HR=0.45, 95% CI 0.32, 0.63). The ALBI score remained independent prognostic value in the validation set for both PFS (*P* = 0.01) and OS (*P* = 0.007). Patients with low ALBI score had better PFS and OS in all subgroups by tumor grade and treatment modalities.

**Conclusions:**

The preoperative ALBI score is a noninvasive and valuable prognostic marker for HGG patients.

## Highlights

• A high ALBI score was associated with poor PFS and OS in HGG patients.
• Low ALBI gourp had 56% decrease of tumor progression risk and 57% decrease of death risk relative to high ALBI patients.
• The prognostic value of ALBI score was externally validated by another cohort of 130 HGG patients.
• ALRI score is an valuable marker for predicting prognosis and guiding clnical management for HGG patients.

## Introduction

High-grade gliomas (HGG), defined as WHO Grade III and IV gliomas, have an incidence of approximately 5 cases per 100,000 individuals, and account for more than 60% of all gliomas [1, 2]. HGG are characterized by high morbidity and mortality owing to their localization and locally invasive nature [3]. Despite aggressive treatment modalities including surgical tumor resection accompanied with fractionated radiotherapy and temozolomide-based chemotherapy were applied, the median survival for glioblastoma (GBM, WHO Grade IV) and anaplastic glioma (WHO Grade III) patients are only 12–14 months and 2–5 years, respectively [4, 5]. Therefore, there is an unmet need to further delineate markers that may provide additional prognostic information and guidance in HGG management.

Increasing evidence indicated a critical role of systemic inflammation in carcinogenesis, cancer proliferation, metastasis and recurrence [6], [7], [8]. Previous studies had shown that increased systemic inflammation of patients correlates with poorer survival in various cancer types, including lung, breast and gastric cancers [9], [10], [11]. Inflammatory related factors including Glasgow Prognostic Score (GPS), neutrophil-lymphocyte ratio (NLR), prognostic nutritional index (PNI) and fibrinogen-albumin (FA) score have been reported as predictors for systemic inflammatory status, as well as the outcomes of patients with HGG [12], [13], [14], [15].

High-grade gliomas are aggressive tumors that display heterogeneous tumor microenvironments with infiltrating immune cells including activated neutrophils, macrophages and lymphocytes [16]. Within the inflammatory microenvironment, reactive oxygen, nitrogen and halogen species released by the activated immune cells may contribute to glioma cell proliferation and tumor invasion [17]. Thus, understanding the systemic inflammatory status of HGG patients may be valuable information for Physicians.

The albumin-bilirubin (ALBI) score has recently been established as a novel evidence-based and easily available tool for the assessment of reserved liver function [18]. More recently, ALBI score was capable of predicting the risk of HCC recurrence following surgical resection [19, 20]. Moreover, ALBI score was reported as a surrogate marker for cancerogenic proinflammatory and immunosuppressive status in hepatocellular carcinoma (HCC) patients [21, 22].However, its prognostic value in the setting of HGG has not yet been defined. Therefore, we conducted a retrospective study to determine the value of preoperative ALBI score for predicting tumor-specific outcomes of HGG patients.

## Patients and methods

### Study population

A total of 331 patients with histologically confirmed as newly diagnosed WHO Grade III and IV gliomas were retrospectively reviewed. All patients were initially treated in Sun Yat-sen University Cancer Center (SYSUCC) from 2001 to 2015. The following inclusion criteria were used: (1) pathologically confirmed diagnosis of WHO Grade III and IV gliomas, (2) no previous malignancy or second primary tumor, (3) no previous anti-tumor treatment before admission, (4) adequate clinical information and followed up data. The exclusion criteria were as follows: (1) patients who had clinical evidence of liver disease, acute infection or chronic active inflammatory diseases, (2) patients who had autoimmune diseases, hematological disorders or anticoagulation treatment, and (3) patients who had perioperative surgery-related mortality. According to these criteria, 7 patients were excluded and 324 patients remained and were analyzed in this study.

Hierarchical group divisible design based on the WHO Grade was applied to dividing the database into training and validation sets. Firstly, random number generated by SPSS software was assigned to all enrolled patients. Secondly, for both WHO Grade III group and WHO Grade IV group, patients were divided into training and validation sets in a ratio of 3:2. Therefore, 194 patients including 93 Grade III patients and 101 Grade IV patients were assigned to the training set. 130 patients including 63 Grade III patients and 67 Grade IV patients were assigned to the validation set. The significance of the tested prognostic values and models identified by the training set were externally validated by the validation set.

To further investigate evaluated the correlation between ALBI score and glioma grade, 72 WHO Grade II glioma patients (54 astrocytoma and 18 oligodendroglioma) and 25 WHO Grade I glioma patients (all pilocytic astrocytoma) were also retrospectively enrolled in this study.

All patients have provided written informed consent for their information to be stored and used in the hospital database. Study approved was obtained from the Medical Ethics Committees of SYSUCC (SZR2020–136) and the study was conducted in accordance with the ethical standard of the World Medical Association Declaration of Helsinki.

### Data collection

The electronic medical record system build in SYSUCC were used to collect baseline characteristics of all enrolled HGG patients. Clinical information including demographics, Karnofsky performance status (KPS), pathological diagnoses, tumor grade, tumor size, tumor location, extent of resection, molecular status, preoperative blood test results and adjuvant treatment regimen were collected. Pathologists in SYSUCC pathology department had reviewed and reclassified all the pathological specimens according to WHO classification (revised in 2016) of central nervous system (CNS) tumors [23]. High-grade gliomas were defined as WHO Grade III and IV gliomas. Tumor location was categorized as cerebral cortex area and non-cerebral cortex area. Tumor size was defined as the maximum diameter measured on preoperative enhanced T1-weighted MRI.

### Therapeutic regimen

All patients enrolled in this study were clinically diagnosed as high-grade glioma after admission. Thus, all enrolled patients underwent surgery in order to obtain pathological diagnosis and achieve maximum safe resection of tumor. The extent of resection was classified as gross total resection (GTR), subtotal resection (STR), partial resection (PR) and biopsy according to postoperative MRI/CT scans and operation notes. Adjuvant radiotherapy and chemotherapy were recommended after histologically confirmed of WHO Grade III or WHO Grade IV gliomas. Adjuvant regimen were defined as fractionated radiotherapy plus first-line chemotherapy or chemo-radiotherapy plus first-line chemotherapy following tumor resection. However, due to poor general condition, refuse of adjuvant therapies or financial problems, part of patients underwent only radiotherapy or only chemotherapy or no adjuvant treatment after tumor resection.

### IDH1 mutation status and other molecular markers

The status of IDH1 mutation was evaluated by polymerase chain reaction (PCR) amplification or immunohistochemistry (IHC) staining (R132H), retrospectively. For PCR analysis, formalin-fixed paraffin-embedded tissue blocks were reviewed for quality control and regions containing more than 50% malignant cells were selected for macrodissection. Genomic DNA was extracted using the QIAamp DNA FFPE Tissue kit (Qiagen, German). The exons 4 of IDH1 was directly sequenced using the Big Dye Terminator v3.1 Cycle Sequencing kit (Applied Biosystems, Foster City, CA) in accordance with the manufacturer's instructions for the 3500XL Genetic Analyzer. All mutations were identified on both strands. The PCR mixture consisted 1200 nmol/primer, 200 nmol/probe, and Taqman Universal PCR Master Mix (PE Applied Biosystems, Foster City, CA) to a final volume of 25μl. Cycling conditions were 50 °C for 2 min and 95 °C for 10 min and followed by 35 cycles at 95 °C for 15 s and 62 °C for 1 min. The primers used for IDH1 exons 4 were TGTGTTGAGATGGACGCCTATTTG (forward) and TGCCACCAACGACCAAGTCA (recerse)

For IHC staining, Paraffin-embedded samples were sectioned (4 μm) and fixed on glass slides. Epitope retrieval was performed in Retrieval solution (ZLI-9607, Golden Bridge, Beijing, China) at pH8.0 heated in a microwave. Slides were subsequently incubated with the primary antibody (mouse anti-IDH1 R132H, 1:100, MAB-0733, MXB Biotechnologies, China) at 4 °C overnight. Antibodies were detected using the substrate diaminobenzidine (DAB, Golden Bridge, Beijing, China).

Expression data of other important molecular markers including p53, O^6^-methylguanine-DNA methyltransferase (MGMT), Epidermal Growth Factor Receptor (EGFR) and Ki-67 were retrieved from the pathological report.

### Inflammatory related markers

Preoperative serum albumin (g/L), total bilirubin (μmol/L), absolute neutrophil counts (10^9^/L), absolute lymphocyte count (10^9^/L), fibrinogen (g/L), C-reactive protein (CRP, mg/L) levels of all enrolled patients were collected. According to the previous study [18], the ALBI score was calculated using serum albumin and bilirubin values according to the introduced formula: 0.66 × log_10_ (total bilirubin μmol/L) −0.085 × (albumin g/L). NLR was calculated by dividing absolute neutrophil counts with absolute lymphocyte count [13]. FA score was determined by the fibrinogen level and albumin level based on previous report [15].

### Follow up

For all patients, follow-up started from the date of operation. Patients were generally followed up quarterly for the first year, semiannually for the following 2 years and annually thereafter. On follow-up, patents will be suggested to have repeat scan of contrast MR-imaging. Recording of medical history, physical examination, and contrast-enhanced MRI scans were routinely performed. The last follow up included verification of the clinical attendance records and direct telecommunication with the patient or their families. Overall survival (OS) was measured from the date of operation to the date of death from any cause or the date of last follow-up visit. Progression-free survival (PFS) was calculated from operation to the first progression, relapse, death from any cause, or the date of the last follow-up visit. Progression or relapse was identified according to the latest radiographic evidence.

### Statistical analysis

Differences of baseline and clinicopathological parameters between groups were evaluated by chi-square test or Fisher's exact test based on the specific type of data. Student's t-test was used to compare ALBI score between different grades. Receiver operating characteristic (ROC) curve was used to determine the optimal cut-off value for the ALBI score. The survival curves were calculated by Kaplan-Meier method and differences between the survival curves were analyzed by log-rank test. All significant parameters, identified by univariate analysis, were further evaluated by multivariate analysis using the Cox proportional hazards model. All reported *P*-values were two-sided. A *P*<0.05 was considered statistically significant. All analyses were carried out using the SPSS 26.0 (IBM Corp., Armonk, NY, United States).

## Results

### Clinico-pathological characteristics of patients in the training set

Table 1 showed the baseline characteristics of 194 patients in the training set. The median age for these patients was 44.0 years (range, 9–78 years), and 65.5% of these patients were males. The training set included 93 (47.9%) WHO Grade III glioma patients and 101 (52.1%) Grade IV glioma patients. In the group of Grade III glioma patients, 65 (69.9%) of patients were pathologically diagnosed as anaplastic astrocytoma, 19 (20.4%) were anaplastic oligodendroglioma, 8 (8.6%) were anaplastic oligoastrocytoma and 1 (1.1%) were gliomatosiscerebri. While, in the group of patients with grade IV gliomas, nearly all of them (100, 99%) were glioblastoma, except 1 (1%) patient was gliosarcoma.

**Table 1.** The clinicopathological features stratified by preoperative ALBI level in the training set (*N* = 194.

Variables	N (%)	ALBI-low, N(%)	ALBI-high, N(%)	*P*
Age, years				0.001
<60	156 (80.4)	90 (57.7)	66 (42.3)	
≥ 60	38 (19.6)	10 (26.3)	28 (73.7)	
Gender				0.889
Male	127 (65.5)	65 (51.2)	62 (48.8)	
Female	67 (34.5)	35 (52.2)	32 (47.8)	
KPS				0.453
≥ 70	184 (94.8)	96 (52.2)	88 (47.8)	
<70	10 (5.2)	4 (40.0)	6 (60.0)	
BMI				0.815
<25 kg/m^2^	150 (77.3)	78 (52.0)	72 (48.0)	
≥ 25 kg/m^2^	44 (22.7)	22(50.0)	22(50.0)	
Tumor grade				0.145
WHO Ⅲ	93 (47.9)	53 (57.0)	40 (43.0)	
WHO Ⅳ	101 (52.1)	47 (46.5)	54 (53.5)	
Tumor size				0.774
≤5 cm	97 (50)	51 (52.6)	46 (47.4)	
>5 cm	97 (50)	49 (50.5)	48 (49.5)	
Tumor location				0.012
Cerebral cortex	174 (90.2)	85 (48.6)	90 (51.4)	
Non cerebral cortex	19 (9.8)	15 (78.9)	4 (21.1)	
Extent of resection				0.692
GTR	124 (63.9)	63 (50.8)	61 (49.2)	
STR	52 (26.8)	26 (50.0)	26 (50.0)	
PR and biopsy	18 (9.3)	11 (61.1)	7 (38.9)	
IDH1 R132H mutation status*			0.844	
Positive	21 (18.3)	11 (52.4)	10 (47.6)	
Negative	94 (81.7)	47 (50.0)	47 (50.0)	
Adjuvant radiotherapy and chemotherapy			0.016	
Yes	114 (58.8)	67 (58.8)	47 (41.2)	
No	80 (41.2)	33 (41.3)	47 (58.7)	

ALBI, albumin-bilirubin score; N, number; KPS, Karnofsky performance status; BMI, body mass index;

WHO, World Health Organization; GTR, Gross total resection; STR, Subtotal resection; PR, Partial resection; IDH1, Isocitrate dehydrogenase 1.

⁎ IDH1 mutation status were only available for 115 patients in the training set.

Gross total resection was achieved in nearly two thirds of all patients (124/194, 63.9%), with 26.8% (52/194) of patients underwent subtotal resection, and 9.2% of patients received partial resection (16/194, 8.2%) or biopsy (2/194, 1%) only. Details of adjuvant treatment modalities were described in Table S1.

### Cut-off determination of ALBI score and its associations with clinicopathological features in the training set

The mean (±SD) value of serum albumin (g/L) and total bilirubin (μmol/L) was 43.20 ± 0.28 g/L and 12.87 ± 0.42μmol/L, respectively. The mean (±SD) value of ALBI score was −2.967 ± 0.024 (range, −4.881 to −2.045). Using overall survival as endpoint, ROC analysis showed that the area under curve (AUC) for ALBI was 0.680 with a 95% CI of 0.598–0.763 (*P*<0.01) (Fig. S1). Accuracy was maximized when ALBI was −2.941, with a sensitivity of 55.8% and a specificity of 74.5%. Therefore, the optimal cutoff value of ABLI was determined as −2.941.

Based on the optimal cutoff value, 100 (100/194, 51.5%) patients were divided into ALBI-low group, and the remaining 94 (48.5%) patients were categorized into ALBI-high group. As shown in Table 1, ALBI level was significantly correlated with age (*P* = 0.001) tumor location (*P* = 0.012) and adjuvant radiotherapy and chemotherapy (*P* = 0.016). Patients with high ALBI level significantly older than patients in ALBI-low group. And patients in the ALBI-high group were more likely to received adjuvant therapies. Other clinical features including age, gender, KPS, BMI, tumor grade, tumor size, extent of resection and IDH1 mutation status were similar between the two groups.

### Prognostic factors influencing survival in the training set

**Fig. 1:**
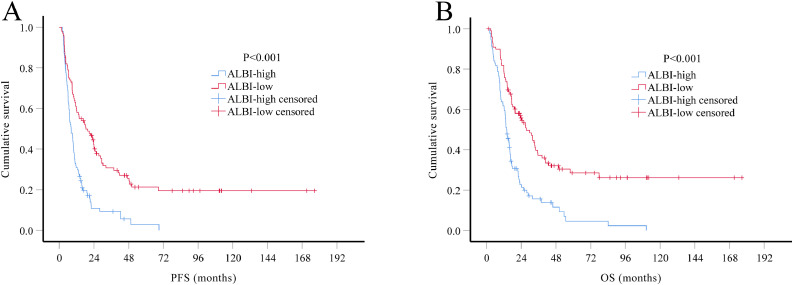
. Kaplan-Meier survival curves of HGG patients in the training set (*N* = 194). According to the optimal cutoff value of ALBI score, patients were divided into two groups: preoperative ALBI score ≤ −2.941 as ALBI-low group and preoperative ALBI score > −2.941 as ALBI-high group. Both PFS (A) and OS (B) of patients in ALBI-low group were better than those in ALBI-high group (both *P*<0.001).

At the date of the last follow-up, 147 (75.8%) patients in the training set had died, with median OS of 17.37 months (95% CI, 14.74–19.99 months). Median OS of grade III patients was 25.53 months (95% CI 13.76–37.30 months) and 14.33 months (95% CI 12.25–16.42 months) of the grade IV patients. Kaplan-Meier analysis showed that, compared with ALBI-low group, both median PFS (8.27 vs. 18.40 months, *P*<0.001) and median OS (13.93 vs. 27.57 months, *P*<0.001) were significantly worse in the ALBI-high group (Fig. 1). Patients in ALBI-low group had better 2-year PFS rates (41.2% vs. 9.3%, *P*<0.001) and 2-year OS rate (54.6% vs. 21.3%, *P*<0.001) than patients in the ALBI-high group.

Univariate analysis showed that ALBI score, as well as traditionally indicators including age, KPS, tumor grade, extent of resection, adjuvant therapy were significant predictors for PFS and OS (Table 2). Strikingly, univariate analysis also demonstrated that patients in ALBI-low group had 56% decrease in the rate of tumor progression (HR 0.44; 95%CI 0.32–0.61) and 57% decrease in the rate of death (HR 0.43; 95%CI 0.31–0.60), compare with those in the ALBI-high group. Multivariate Cox regression analysis including all significant factor in univariate analysis showed that ALBI score, age, tumor grade, extent of resection, adjuvant therapy remained as significant independent predictors for both PFS and OS in the training set (Table 2).

**Table 2.** The univariate and multivariate analysis of the prognostic factors for PFS and OS in the training set (*N* = 194).

		PFS				OS			
Variables	Number	Univariate analysis		Multivariate analysis		Univariate analysis		Multivariate analysis	
		HR (95% CI)	*P* value	HR (95% CI)	*P* value	HR (95% CI)	*P* value	HR (95% CI)	*P* value
Age, years			<0.001		0.009		<0.001		0.032
<60/≥60	156/38	2.38 (1.63–3.49)		1.74 (1.15–2.64)		2.25 (1.52–3.32)		1.60 (1.04–2.46)	
Gender			0.063				0.067		
Male/ Female	127/67	0.73 (0.52–1.02)			–	0.72 (0.51–1.02)		–	–
KPS			0.011		0.296		0.009		0.317
≥70/<70	184/10	2.33 (1.22–4.47)		1.47 (0.72–3.00)		2.39 (1.24–4.57)		1.44 (0.71–2.94)	
BMI (Kg/m^2^)			0.508				0.673		
<25.0/≥25.0	150/44	0.88 (0.61–1.28)		–	–	0.92 (0.62–1.38)		–	–
Tumor grade			0.002		0.006		0.001		0.003
Ⅲ/Ⅳ	93/101	1.66 (1.21–2.27)		1.59 (1.14–2.22)		1.74 (1.25–2.42)		1.69 (1.19–2.39)	
Tumor size (cm)			0.867				0.608		
≤5/>5	97/97	0.97 (0.71–1.33)		–	–	0.92 (0.66–1.27)		–	–
Extent of resection			<0.001		<0.001		<0.001		<0.001
GTR	124	1 (Referent)		1 (Referent)		1 (Referent)		1 (Referent)	
STR	58	1.53 (1.08–2.17)	0.017	1.75 (1.22–2.52)	0.003	1.53 (1.06–2.19)	0.022	1.80 (1.22–2.64)	0.003
PR or biopsy	18	3.29 (1.95–5.54)	<0.001	4.83 (2.77–8.43)	<0.001	3.04 (1.78–5.21)	<0.001	4.68 (2.64–8.29)	<0.001
ALBI score			<0.001		<0.001		<0.001		<0.001
High/Low	94/100	0.44 (0.32–0.61)		0.47 (0.34–0.66)		0.43 (0.31–0.60)		0.45 (0.32–0.63)	
Adjuvant radiotherapy and chemotherapy			0.002		0.004		<0.001		<0.001
Yes/No	114/80	1.65 (1.20–2.25)		1.62 (1.17–2.24)		1.95 (1.41–2.70)		1.96 (1.41–2.74)	

PFS, progression-free survival; OS, overall survival; HR, hazard ratio; CI, confidence interval; KPS, Karnofsky performance status; GTR, Gross total resection; STR, Subtotal resection; PR, Partial resection; ALBI, Albumin- bilirubin.

### Prognostic factors influencing survival in the validation set

An independent set of consecutive, demographic-matched 130 HGG patients was used to statistically validate the prognostic model identified in the training set. There was no significant difference between the training and validation sets with regards to age, gender, KPS, BMI, tumor grade, tumor size, extent of resection, IDH1 mutation status and treatment modalities (all *P* > 0.05). Both median PFS (10.60 vs. 10.50 months, *P* = 0.797) and OS (16.90 vs. 17.37 months, *P* = 0.566) in the validation set were similar with those in the training set.

Multivariate analysis in the validation set confirmed that ALBI score still was an independent predictor for both PFS (HR 0.59, 95% CI 0.39–0.88) and OS (HR 0.56, 95% CI 0.36–0.85) (Table 3).

**Table 3.** The multivariate analysis of the prognostic factors for PFS and OS in validation set (*N* = 130).

		PFS			OS		
Variables	Number	HR	95% CI	P value	HR	95% CI	P value
Age, years				0.033			0.046
<60/≥60	111/19	1.77	1.05–3.00		1.65	0.99–2.73	
KPS				0.800			0.852
≥70/<70	117/13	1.08	0.58–2.02		1.06	0.57–1.96	
Tumor grade				0.001			<0.001
Ⅲ/Ⅳ	63/67	2.11	1.38–3.21		2.52	1.62–3.90	
Extent of resection				<0.001			<0.001
GTR	81	1	Referent		1	Referent	
STR	37	1.79	1.15–2.77	0.010	1.57	1.00–2.48	0.051
PR or biopsy	12	6.80	3.28–14.1	<0.001	4.96	2.43–10.1	<0.001
ALBI score				0.010			0.007
High/Low	48/82	0.59	0.39–0.88		0.56	0.36–0.85	
Adjuvant radiotherapy and chemotherapy				<0.001			<0.001
Yes/No	69/61	2.36	1.60–3.50		2.85	1.90–4.28	

PFS, progression-free survival; OS, overall survival; HR, hazard ratio; CI, confidence interval; KPS, Karnofsky performance status; GTR, Gross total resection; STR, Subtotal resection; PR, Partial resection; ALBI, Albumin- bilirubin.

### Multivariate Cox regression analysis of the role of ALBI score in predicting OS and PFS in the combination of two sets

**Fig. 2:**
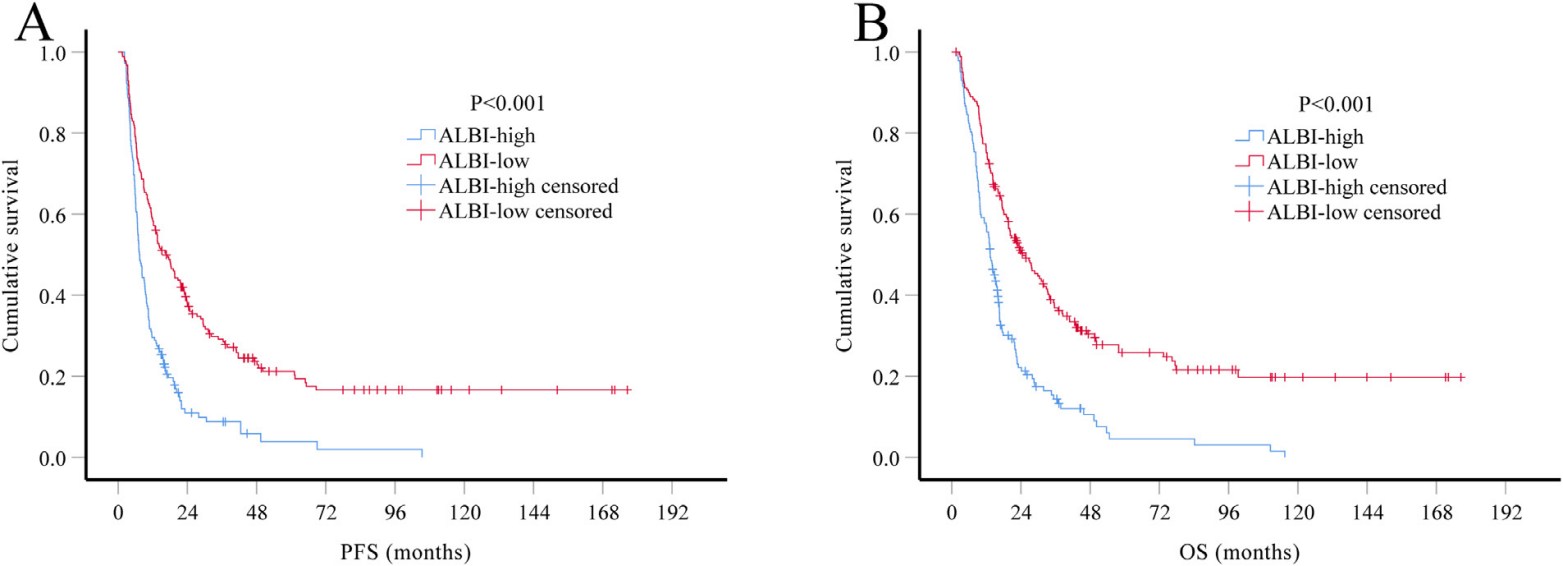
. Kaplan-Meier survival curves of all HGG patients (*N* = 324). Patients in ALBI-low group had significantly longer PFS (A, *P*<0.001) and OS (B, *P*<0.001) than patients in ALBI-high group.

The prognostic value of ALBI score was further evaluated in all HGG patients combined both training and validation sets (*n* = 324). Multivariate Cox regression analysis demonstrated that ALBI score is still one of the most powerful indicator for both PFS (HR 0.52, 95% CI 0.40–0.67) and OS (HR 0.51, 95% CI 0.39–0.66), independent of age, tumor grade, extent of resection and adjuvant therapy (Table 4; Fig. 2).

**Table 4.** The multivariate analysis of the prognostic factors for PFS and OS in all patients (*N* = 324).

		PFS			OS		
Variables	Number	HR	95% CI	*P* value	HR	95% CI	*P* value
Age, years				0.001			0.003
<60/≥60	267/57	1.73	1.26–2.38		1.65	1.19–2.29	
KPS				0.207			0.236
≥70/<70	301/23	1.34	0.85–2.12		1.32	0.83–2.08	
Tumor grade				<0.001			<0.001
Ⅲ/Ⅳ	156/168	1.72	1.34–2.22		1.85	1.42–2.41	
Extent of resection				<0.001			<0.001
GTR	205	1	Referent		1	Referent	
STR	89	1.79	1.35–2.36	<0.001	1.71	1.28–2.29	<0.001
PR	30	5.23	3.40–8.04	<0.001	5.24	3.37–8.14	<0.001
ALBI score				<0.001			<0.001
High/Low	142/182	0.52	0.40–0.67		0.51	0.39–0.66	
Adjuvant radiotherapy and chemotherapy				<0.001			<0.001
Yes/No	173/145	1.85	1.45–2.36		2.23	1.73–2.87	

PFS, progression-free survival; OS, overall survival; HR, hazard ratio; CI, confidence interval; KPS, Karnofsky performance status; GTR, Gross total resection; STR, Subtotal resection; PR, Partial resection; ALBI, Albumin-bilirubin.

**Fig. 3:**
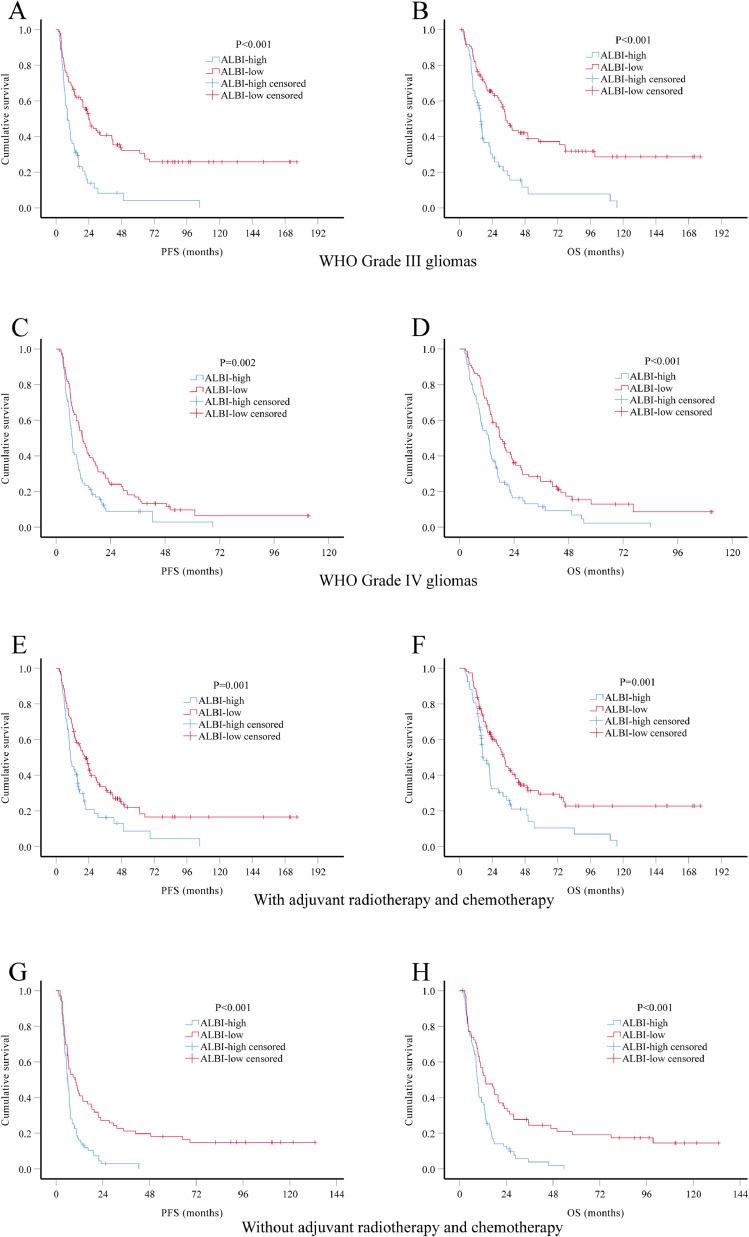
. Kaplan-Meier survival curves of different HGG subgroups. Kaplan-Meier method and log-rank test were used to investigate differences in PFS and OS by preoperative ALBI group. Low ABLI level was significantly associated with better PFS and OS in subgroups of WHO Grade III gliomas (A-B), WHO Grade IV gliomas (C-D), patients received adjuvant radiotherapy and chemotherapy (E-F) and patients did not receive adjuvant radiotherapy and chemotherapy (G-H).

Considering the impact of tumor grade and adjuvant therapy in the prognosis of HGG patients, we performed subgroup analysis stratified by these two factors. Again, both PFS and OS were significantly better in ALBI-low group in all subgroup analysis (Fig. 3). We also performed multivariate Cox regression analysis in patients with known IDH1 mutation status (*n* = 196). Results showed that ALBI score remain a significant prognostic factor for both PFS (HR 0.55, 95% CI 0.40–0.74) and OS (HR 0.53, 95% CI 0.39–0.73), independent of age, extent of resection, IDH1 mutation and adjuvant therapy (Table 5).

**Table 5.** The multivariate analysis of prognostic factors for PFS and OS in patients with IDH1 status (*N* = 196).

		PFS			OS		
Variables	Number	HR	95% CI	P value	HR	95% CI	P value
Age, years				0.002			<0.001
<60/≥60	157/39	1.81	1.24–2.66		2.01	1.34–2.97	
KPS				0.054			0.097
≥70/<70	181/15	1.77	0.99–3.15		1.63	0.92–2.90	
Tumor grade				0.039			0.024
Ⅲ/Ⅳ	75/121	1.43	1.02–2.00		1.50	1.06–2.14	
Extent of resection				<0.001			<0.001
GTR	121	1	Referent		1	Referent	
STR	57	1.88	1.31–2.70	0.001	1.74	1.20–2.52	0.003
PR	18	6.64	3.81–11.6	<0.001	5.34	3.03–9.39	<0.001
ALBI score				<0.001			<0.001
High/Low	110/86	0.55	0.40–0.75		0.53	0.39–0.73	
Adjuvant radiotherapy and chemotherapy				<0.001			<0.001
Yes/No	105/91	2.01	1.46–2.78		2.52	1.82–3.50	
IDH1 status				0.017			0.035
Positive/Negative	32/164	1.76	1.11–2.82		1.68	1.04–2.71	

PFS, progression-free survival; OS, overall survival; HR, hazard ratio; CI, confidence interval; KPS, Karnofsky performance status; GTR, Gross total resection; STR, Subtotal resection; PR, Partial resection; ALBI, Albumin- bilirubin; IDH1, isocitrate dehydrogenase 1.

### Correlations between ALBI score and other factors

To further investigate the correlations of ALBI score and other inflammatory markers, Chi-square tests were performed for ALBI score, NLR, FA score and CRP level in all enrolled patients. Resulte showed while ABLI score was not correlated with NLR (*P* = 0.416), it was positively correlated with FA score (*P* < 0.001) and CRP level (*P* = 0.025) (Table S2).

To further identified if ALBI score could serve as a predictive factor for pathological grade of glioma patients, we retrospectively enrolled 72 WHO Grade II glioma patients (54 astrocytoma and 18 oligodendroglioma) and 25 WHO Grade I glioma patients (all pilocytic astrocytoma) into this study. Student's t tests were used to compare ALBI score between different grades. Results showed that Grade IV glioma patients had significantly higher ALBI score than WHO Grade III (*P* = 0.020) and WHO Grade I (*P* = 0.017) patients, except for WHO Grade II patients (*P* = 0.218) (Fig. S2).

Expression of other molecular markers including p53, MGMT, EGFR and Ki-67 are also important for HGG patient. Therefore, Chi-square test or Fisher's exact test was used to further evaluate the potential correlations between ALBI score and the molecular markers. Results showed that ALBI score is only significantly correlated with Ki-67 expression level (*P* = 0.009). Patients with low Ki-67 expression level tend to have low ALBI score too. No Significant correlation was identified between ALBI score and other markers including p53, EGFR, MGMT and IDH1 mutation (Table S3).

## Discussion

The ALBI score was originally developed to assess the liver function in patients with hepatocellular carcinoma (HCC) before tumor resection [18]. Compared with other liver functional reserve model, ALBI grade showed superior prognostic power in predicting postoperative liver failure and survival in HCC patients receiving surgical resection [24]. Several studies held by clinical investigators also reported that, other than predicting reserved liver function, ALBI score can significantly predict the risk of HCC recurrence in different groups of patients receiving therapeutic approaches including surgery, radiotherapy, target therapy or liver transplantation [19, 22, 25, 26].

Though ALBI was initially purposed for HCC patients, recent studies also suggested that preoperative ALBI grade hold its prognostic power in gastric cancer or pancreatic cancer patients [27, 28]. Apart from that, all of these studied finally emphasized that ALBI score reflects not only the liver function, but also the pro-tumor inflammatory and immunosuppressive status [10, 22, 25, 28]. Moreover, several systemic inflammatory markers such as GPS, NLR, PNI, were reported as prognostic indexes in HGG patients [12], [13], [14]. Therefore, we planned to investigate the potential prognostic value of ALBI score in our cohorts of HGG patients.

Here in this study we demonstrated that preoperative ALBI score was a valuable biomarker for predicting survival in patients with HGG after resection. Using −2.941 as the optimal cutoff value, patient with high ALBI score had significantly worse PFS and OS than those in low ALBI group. Multivariate analysis showed low ALBI score was an independent indicator for better PFS and OS in the training set. The prognostic value of ALBI score was externally validated by another cohort of HGG patients. Thus, the preoperative ALBI is noninvasive and promising predictor, which could be potentially applied to identify HGG patients at an increased risk of progression or death.

Further investigation about the correlations between ALBI score and other factors revealed that ALBI score correlated with FA score (*P* < 0.001) and CRP level (*P* = 0.025). The reason of significant correlation between ABLI score and FA score is mainly the same albumin parameter was used for calculating both score. And patients with high ALBI score tend to have high CRP level as well. Since only a portion of patients had CRP data, we may need larger sample size to confirm the actual correlation status between ALBI and CRP. We also found out ALBI is significantly correlated with Ki-67 expression level (*P* = 0.009). We speculated that HGG patients with low systemic inflammatory level, which was reflected by low ABLI score, had less reactive oxygen, nitrogen and halogen species within the microenvironment. Therefore, these patients might also have a less proliferative tumor with lower Ki-67 expression and longer survival.

Further analysis between ABLI score and glioma grade demonstrated preoperative ALBI score may be a potentially valuable factor for differentiating Grade IV glioma from other lower grade gliomas. Yet data from healthy controls and other CNS tumor like meningioma and acoustic neuroma are needed for further investigation.

Although the prognostic value of ABLI score has been reported in patients with various cancers [10, 19, 28], the mechanism remains elusive. Regarding to the predictive value of ALBI in HGG patients, we speculated that it mainly relies on the reflection of systemic inflammatory and immunosuppressive status. Serum albumin is a valuable indicator of host nutritional status and systemic inflammatory response [29, 30]. Several studies already demonstrated that albumin level alone can predict outcomes across various types of cancer [30, 31, –32]. Hypoalbuminemia, which reflects malnutrition, also correlates with elevated systemic inflammatory markers [33]. Pro-inflammatory cytokines such as interleukin-1, interleukin-6, tumor necrosis factor α, are not only important impact factors for albumin production [34], but also critical in oncogenesis, angiogenesis and tumor progression [35], [36], [37].

Bilirubin was originally considered as a biomarker for liver disease. However, recent data suggested endogenous bilirubin is also a powerful signaling molecule [38, 39]. Unconjugated bilirubin has been recognized as a potent activator of the aryl hydrocarbon receptor (AhR) [40, 41]. AhR has an important role in modulating differentiation processes of regulatory T cells (Treg), T-helper 17 cells (Th17) and B cells [42], [43], [44]. AhR regulates the transcription and epigenetic status of the Treg master transcription factor FoxP3 in FoxP3^+^ Tregs [45]. Other than the critical roles in immune cells, AhR also has a role in astrocyte related inflammatory response. Rothhammer et al. found that type-I interferons (IFN-Is) induce AhR expression in astrocytes, triggering AhR-dependent anti-inflammatory transcriptional responses [46]. Moreover, AhR agonists like bilirubin can reached the central nerve system (CNS) and activated this anti-inflammatory response in astrocytes [42]. This phenomenon might explain the general immunosuppressive effects of bilirubin. Therefore, these studies can help explain the prognostic role of ALBI for the patients with HGG.

As with most retrospective studies, our study had several limitations. First, ROC analysis revealed that when accuracy was maximized, ALBI score only had a sensitivity of 55.8% and a specificity of 74.5%. Though specificity is relatively high, sensitivity is not good enough. Further study is needed to search other valuable inflammatory factors that could be integrated with ALBI to raise the sensitivity and specificity. Secondly, this is a single-center designed retrospective analysis with a moderate sample size, which may suffer from selection bias. Thirdly, important molecular characteristic data including TERT, 1p/19q LOH and ATRX were unobtainable because of tumor tissue status. Fourthly, because of its retrospective nature, information of PFS might not be exactly accurate in the present study. Despite its preliminary feature, this study showed convincing prognostic value of ALBI score for HGG patients. However, more prospective trials are needed to validate our findings and determine the optimal ALBI score calculation for HGG patients.

## Conclusions

In conclusion, our present study demonstrated that the preoperative ALBI score is an independent prognostic factor for predicting the PFS and OS for patients with HGG. Patients with low ALBI score had better PFS and OS than those patients with higher ALBI score. ALRI score is an easily accessible, noninvasive marker that may provide immense help in predicting prognosis and guiding the individualized treatment for HGG patients.

## Declaration of Competing Interest

The authors report no conflicts of interest in this work.

## Appendix Supplementary materials

Image, application 1

Image, application 2

## References

[1] Ostrom Q.T., Gittleman H., Truitt G. (2018). CBTRUS statistical report: primary brain and other central nervous system tumors diagnosed in the United States in 2011-2015. Neuro Oncol..

[2] Molinaro A.M., Taylor J.W., Wiencke J.K. (2019). Genetic and molecular epidemiology of adult diffuse glioma. Nat. Rev. Neurol..

[3] Cuddapah V.A., Robel S., Watkins S. (2014). A neurocentric perspective on glioma invasion. Nat. Rev. Neurosci..

[4] Ostrom Q.T., Cote D.J., Ascha M. (2018). Adult glioma incidence and survival by race or ethnicity in the United States from 2000 to 2014. JAMA Oncol..

[5] Gilbert M.R., Dignam J.J., Armstrong T.S. (2014). A randomized trial of bevacizumab for newly diagnosed glioblastoma. N. Engl. J. Med..

[6] Greten F.R., Grivennikov S.I. (2019). Inflammation and cancer: triggers, mechanisms, and consequences. Immunity.

[7] Albrengues J., Shields M.A., Ng D. (2018). Neutrophil extracellular traps produced during inflammation awaken dormant cancer cells in mice. Science.

[8] Zitvogel L., Pietrocola F., Kroemer G. (2017). Nutrition, inflammation and cancer. Nat. Immunol..

[9] Wang K., Sun J.Z., Wu Q.X. (2020). Long-term anti-inflammatory diet in relation to improved breast cancer prognosis: a prospective cohort study. NPJ Breast Cancer.

[10] Lin J.X., Lin J.P., Xie J.W. (2019). Prognostic importance of the preoperative modified systemic inflammation score for patients with gastric cancer. Gastric Cancer.

[11] Gao Y., Zhang H., Li Y. (2018). Preoperative increased systemic immune-inflammation index predicts poor prognosis in patients with operable non-small cell lung cancer. Clin. Chim. Acta.

[12] Topkan E., Selek U., Ozdemir Y. (2018). Prognostic value of the glasgow prognostic score for glioblastoma multiforme patients treated with radiotherapy and temozolomide. J. Neurooncol..

[13] Weng W., Chen X., Gong S. (2018). Preoperative neutrophil-lymphocyte ratio correlated with glioma grading and glioblastoma survival. Neurol. Res..

[14] He Z.-.Q., Ke C., Al-Nahari F. (2017). Low preoperative prognostic nutritional index predicts poor survival in patients with newly diagnosed high-grade gliomas. J. Neurooncol..

[15] He Z.-.Q., Duan H., Ke C. (2017). Evaluation of cumulative prognostic score based on pretreatment plasma fibrinogen and serum albumin levels in patients with newly diagnosed high-grade gliomas. Oncotarget.

[16] Ma Q., Long W., Xing C. (2018). Cancer stem cells and immunosuppressive microenvironment in glioma. Front. Immunol..

[17] Sowers J.L., Johnson K.M., Conrad C. (2014). The role of inflammation in brain cancer. Adv. Exp. Med. Biol..

[18] Johnson P.J., Berhane S., Kagebayashi C. (2015). Assessment of liver function in patients with hepatocellular carcinoma: a new evidence-based approach-the ALBI grade. J. Clin. Oncol..

[19] Toyoda H., Lai P.B., O'Beirne J. (2016). Long-term impact of liver function on curative therapy for hepatocellular carcinoma: application of the ALBI grade. Br. J. Cancer.

[20] Ho S.Y., Hsu C.Y., Liu P.H. (2019). Albumin-bilirubin (ALBI) grade-based nomogram to predict tumor recurrence in patients with hepatocellular carcinoma. Eur. J. Surg. Oncol..

[21] Casadei Gardini A., Foschi F.G., Conti F. (2019). Immune inflammation indicators and ALBI score to predict liver cancer in HCV-patients treated with direct-acting antivirals. Dig. Liver Dis..

[22] Kornberg A., Witt U., Schernhammer M. (2019). The role of preoperative albumin-bilirubin grade for oncological risk stratification in liver transplant patients with hepatocellular carcinoma. J. Surg. Oncol..

[23] Louis D.N., Perry A., Reifenberger G. (2016). The 2016 World Health Organization classification of tumors of the central nervous system: a summary. Acta Neuropathol..

[24] Ho S.Y., Liu P.H., Hsu C.Y. (2018). Comparison of twelve liver functional reserve models for outcome prediction in patients with hepatocellular carcinoma undergoing surgical resection. Sci. Rep..

[25] Tada T., Kumada T., Toyoda H. (2019). Impact of albumin-bilirubin grade on survival in patients with hepatocellular carcinoma who received sorafenib: an analysis using time-dependent receiver operating characteristic. J. Gastroenterol. Hepatol..

[26] Ho C.H.M., Chiang C.L., Lee F.A.S. (2018). Comparison of platelet-albumin-bilirubin (PALBI), albumin-bilirubin (ALBI), and child-pugh (CP) score for predicting of survival in advanced hcc patients receiving radiotherapy (RT). Oncotarget.

[27] Kanda M., Tanaka C., Kobayashi D. (2018). Preoperative albumin-bilirubin grade predicts recurrences after radical gastrectomy in patients with pT2-4 gastric cancer. World J. Surg..

[28] Yagyu T., Saito H., Sakamoto T. (2019). Preoperative albumin-bilirubin grade as a useful prognostic indicator in patients with pancreatic cancer. Anticancer Res..

[29] Sánchez-Lara K., Turcott J.G., Juárez E. (2012). Association of nutrition parameters including bioelectrical impedance and systemic inflammatory response with quality of life and prognosis in patients with advanced non-small-cell lung cancer: a prospective study. Nutr. Cancer.

[30] Borg N., Guilfoyle M.R., Greenberg D.C. (2011). Serum albumin and survival in glioblastoma multiforme. J. Neurooncol..

[31] Gupta D., Lis C.G. (2010). Pretreatment serum albumin as a predictor of cancer survival: a systematic review of the epidemiological literature. Nutr. J..

[32] Ishizuka M., Nagata H., Takagi K. (2016). Clinical significance of the C-reactive protein to albumin ratio for survival after surgery for colorectal cancer. Ann. Surg. Oncol..

[33] Hu W.H., Eisenstein S., Parry L. (2019). Preoperative malnutrition with mild hypoalbuminemia associated with postoperative mortality and morbidity of colorectal cancer: a propensity score matching study. Nutr. J..

[34] Hülshoff A., Schricker T., Elgendy H. (2013). Albumin synthesis in surgical patients. Nutrition.

[35] Weissenberger J., Loeffler S., Kappeler A. (2004). IL-6 is required for glioma development in a mouse model. Oncogene.

[36] Kore R.A., Abraham E.C. (2014). Inflammatory cytokines, interleukin-1 beta and tumor necrosis factor-alpha, upregulated in glioblastoma multiforme, raise the levels of CRYAB in exosomes secreted by U373 glioma cells. Biochem. Biophys. Res. Commun..

[37] Wang Q., He Z., Huang M. (2018). Vascular niche IL-6 induces alternative macrophage activation in glioblastoma through HIF-2alpha. Nat. Commun..

[38] Vitek L. (2020). Bilirubin as a signaling molecule. Med. Res. Rev..

[39] Gazzin S., Vitek L., Watchko J. (2016). A novel perspective on the biology of bilirubin in health and disease. Trends Mol. Med..

[40] Phelan D., Winter G.M., Rogers W.J. (1998). Activation of the Ah receptor signal transduction pathway by bilirubin and biliverdin. Arch. Biochem. Biophys..

[41] Barouki R., Aggerbeck M., Aggerbeck L. (2012). The aryl hydrocarbon receptor system. Drug Metabol. Drug Interact..

[42] Wheeler M.A., Rothhammer V., Quintana F.J. (2017). Control of immune-mediated pathology via the aryl hydrocarbon receptor. J. Biol. Chem..

[43] Gutierrez-Vazquez C., Quintana F.J. (2018). Regulation of the immune response by the aryl hydrocarbon receptor. Immunity.

[44] Longhi M.S., Vuerich M., Kalbasi A. (2017). Bilirubin suppresses Th17 immunity in colitis by upregulating CD39. JCI insight.

[45] Gandhi R., Kumar D., Burns E.J. (2010). Activation of the aryl hydrocarbon receptor induces human type 1 regulatory T cell-like and Foxp3(+) regulatory T cells[J]. Nat. Immunol..

[46] Rothhammer V., Mascanfroni I.D., Bunse L. (2016). Type I interferons and microbial metabolites of tryptophan modulate astrocyte activity and central nervous system inflammation via the aryl hydrocarbon receptor. Nat Med..

