# Supplementary material for: High preoperative albumin-bilirubin score predicts poor survival in patients with newly diagnosed high-grade gliomas
Source: Transl Oncol. 2021 Feb 14;14(4):101038. doi: 10.1016/j.tranon.2021.101038 (PMC7893483; doi:10.1016/j.tranon.2021.101038)
Supplement: Supplementary file 1 [file mmc1.docx]

Table S1 Treatment modality for the patients in training set (N=194).

A, Temozolomide-based; B, nitrosourea-based or platinum-based

| Treatment madality | N (%) |
| --- | --- |
| Surgical resection only | 57 (29.4) |
| Surgery→Radiotherary | 19 (9.8) |
| Surgery→Radiotherary→Chemotheraphy (A) | 80 (41.2) |
| Surgery→Radiotherary→Chemotheraphy (B) | 30 (15.5) |
| Surgery→Chemotheraphy (A) | 4 (2.1) |
| Surgery→Chemotheraphy (B) | 4 (2.1) |

Table S2 Correlations between ALBI score and other inflammatory markers in all enrolled patients.

| Variables | N (%) | ALBI-low, N(%) | ALBI-high, N(%) | *P* |
| --- | --- | --- | --- | --- |
| NLR* |  |  |  | 0.416 |
| ＜4.0 | 185 (58.2) | 100 (54.1) | 85 (58.6) |  |
| ≥ 4.0 | 133 (41.8) | 78 (45.9) | 55 (41.4) |  |
| FA score |  |  |  | ＜0.001 |
| 0 | 79 (24.4) | 78 (98.7) | 1 (1.3) |  |
| 1 | 169 (52.1) | 91 (53.8) | 78 (46.2) |  |
| 2 | 76 (23.5) | 13 (17.1) | 63 (82.9) |  |
| CRP^#^ |  |  |  | 0.025 |
| ＜1 mg/L | 116 (53.0) | 77 (66.4) | 39 (33.6) |  |
| ≥ 1 mg/L | 103 (47.0) | 53 (51.5) | 50 (48.5) |  |

NLR, neutrophil-lymphocyte ratio; FA score, fibrinogen-albumin score; CRP, C-reactive protein; N, number

* NLR data were available in 318 patients.

# CRP data were available in only 219 patients.

Table S3 Correlations between ALBI score and molecular markers in all enrolled patients.

| Markers | N (%) | ALBI-low, N(%) | ALBI-high, N(%) | *P* |
| --- | --- | --- | --- | --- |
| p53 ^a^ | |  |  | 0.850 |
| Positive | 163 (85.3) | 90 (55.2) | 73 (44.8) |  |
| Negative | 28 (14.7) | 16 (57.1) | 12 (42.9) |  |
| EGFR ^b^ |  |  |  | 0.181 |
| Positive | 64 (64.6) | 39 (57.7) | 25 (42.3) |  |
| Negative | 35 (35.4) | 119 (26.3) | 93 (73.7) |  |
| MGMT ^c^ |  |  |  | 0.221 |
| Positive | 126 (57.3) | 80 (63.5) | 46 (36.5) |  |
| Negative | 94 (42.7) | 52 (55.3) | 42 (44.7) |  |
| Ki-67 ^d^ | |  |  | 0.009 |
| 0-9% | 52 (21.7) | 41 (78.8) | 11 (21.2) |  |
| 10-19% | 72 (30.0) | 36 (50.0) | 36 (50.0) |  |
| 20-49% | 91 (37.9) | 50 (54.9) | 41 (45.1) |  |
| 50-100% | 25 (10.4) | 14 (56.0) | 11 (44.0) |  |
| IDH1 R132H mutation ^e^ | |  |  | 0.427 |
| Positive | 32 (53.0) | 20 (62.5) | 12 (37.5) |  |
| Negative | 164 (47.0) | 90 (54.9) | 74 (45.1) |  |

EGFR, Epidermal Growth Factor Receptor; MGMT, O^6^-methylguanine-DNA methyltransferase;

IDH1, Isocitrate dehydrogenase 1

1. p53 expression data were available in 191 patients.
2. EGFR expression data were available in 99 patients.
3. MGMT expression data were available in 220 patients.
4. Ki-67 expression data were available in 240 patients.
5. IDH1 mutation data were available in 196 patients.
