# Supplementary material for: High preoperative albumin-bilirubin score predicts poor survival in patients with newly diagnosed high-grade gliomas
Source: Transl Oncol. 2021 Feb 14;14(4):101038. doi: 10.1016/j.tranon.2021.101038 (PMC7893483; doi:10.1016/j.tranon.2021.101038)

Fig.S1 ROC curve of ALBI score in the training set (N=194).

Receiver operating characteristic (ROC) analysis was performed to evaluate the optimal cut-off value of ALBI score. The area under curve (AUC) for ALBI was 0.680 with a 95% CI of 0.598-0.763 (P<0.001). When ALBI was -2.941, Accuracy was maximized when ALBI was -2.941, with a sensitivity of 55.8% and a specificity of 74.5%.


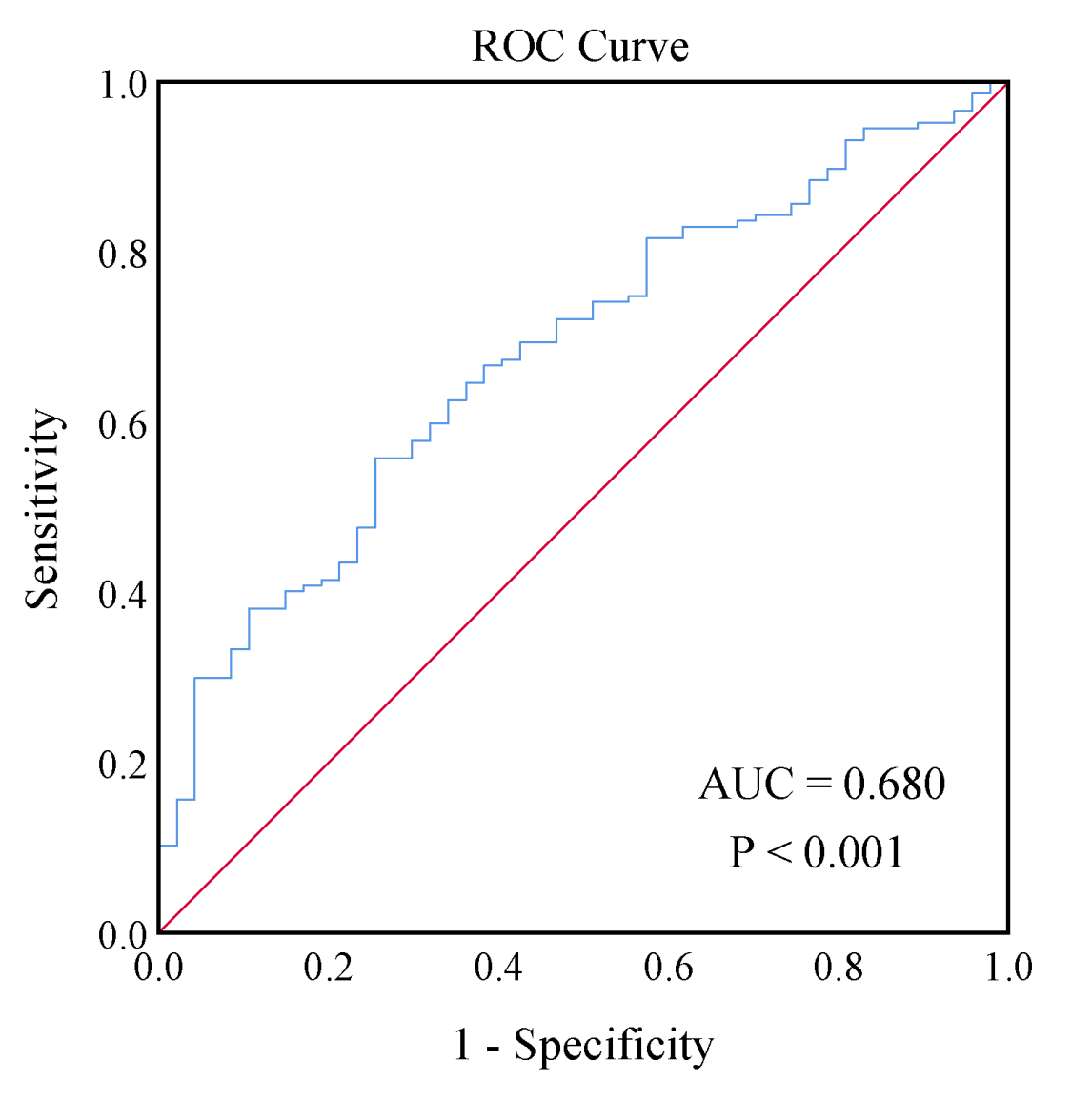


Fig. S2 Box plot of preoperative ALBI score compared with glioma grade.

The boxes and whiskers represent interquartile range and full range, respectively. The data points past the whiskers indicate outliers. WHO Grade IV glioma patients had significantly higher ALBI score than WHO Grade III (P=0.020) and WHO Grade I (P=0.017) patients, except for WHO Grade II patients (P=0.218).


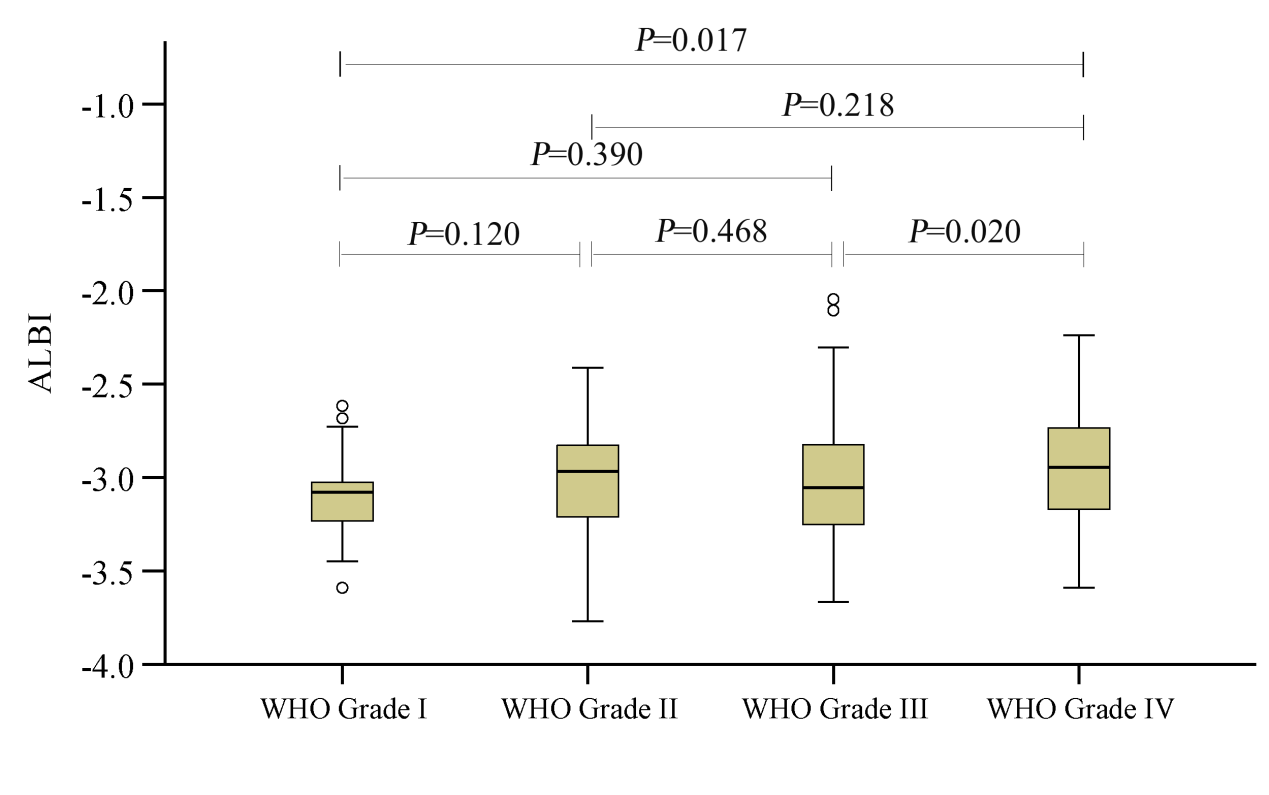

Supplement: Supplementary file 2 [file mmc2.docx]
